# Keratin 80 regulated by miR-206/ETS1 promotes tumor progression via the MEK/ERK pathway in ovarian cancer

**DOI:** 10.7150/jca.64031

**Published:** 2021-09-24

**Authors:** Ouxuan Liu, Caixia Wang, Shuang Wang, Yuexin Hu, Rui Gou, Hui Dong, Siting Li, Xiao Li, Bei Lin

**Affiliations:** 1 Department of Obstetrics and Gynecology, Shengjing Hospital of China Medical University, Liaoning, China.; 2 Key Laboratory of Maternal-Fetal Medicine of Liaoning Province, Key Laboratory of Obstetrics and Gynecology of Higher Education of Liaoning Province, Liaoning, China.; 3 Department of Obstetrics and Gynecology, West China Second University Hospital, Sichuan University, Chengdu, China.

**Keywords:** ovarian cancer, KRT80, MEK/ERK pathway, ETS1, miR-206

## Abstract

**Introduction:** Keratin 80 (KRT80) is a type II epithelial keratin protein that plays an important role in cell differentiation and tumor progression. However, its role and mechanisms in ovarian cancer remain unclear.

**Methods:** The effect of KRT80 on the survival and prognosis of patients with ovarian cancer was determined using immunohistochemistry. Cell lines overexpressing KRT80 and with KRT80 knockdown were established to study its effect on the malignant behavior of ovarian cancer cells. Western blotting was used to detect changes in related molecules, and in the MEK/ERK signal transduction pathway. ChIP assay was used to confirm that ETS1 regulates KRT80 at the transcriptional level. A double luciferase assay was used to confirm the target of miR-206.

**Results:** The expression levels of KRT80 were high in ovarian cancer tissue, and were related to survival and prognosis. KRT80 expression is an independent prognostic factor in patients with ovarian cancer. KRT80 overexpression promotes the proliferation of ovarian cancer cells, the transition from G1 phase to S phase, invasion, and migration. KRT80 overexpression increased the expression of BCL2/BAX, CyclinD1, MMP2, MMP9, and N-cadherin, decreased the expression of E-cadherin, and increased the phosphorylation of MEK and ERK. ETS1 binds to the upstream promoter sequence of KRT80 and regulates KRT80 expression at the transcriptional level. ETS1 is a direct target of miR-206 in ovarian cancer cells.

**Conclusion:** KRT80 regulated by miR-206/ETS1 promotes tumor progression via the MEK/ERK pathway in ovarian cancer, and KRT80 may have applications as a screening biomarker and potential therapeutic target for ovarian cancer.

## Introduction

Ovarian cancer (OC) is one of the most common gynecologic malignancies worldwide, and is the leading cause of death due to gynecologic malignancies 1, 2. The 5 year survival rate for ovarian cancer patients is approximately 47%, whereas the 5 year survival rate for stage III and IV ovarian cancer patients is only 29% 3, 4. Due to a lack of effective and sensitive clinical screening methods for early ovarian cancer, it is urgent to further explore specific tumor markers to predict the occurrence and outcome of ovarian cancer. Therefore, it is vital to search for genes involved in the occurrence, development, and chemotherapy resistance of ovarian cancer, and to explore their applicability in the early diagnosis, disease monitoring, and prognosis evaluation of ovarian cancer.

The *KRT80* gene is located at the centromeric end of the human type II keratin gene domain on chromosome 12q13.13, and encodes a 452-amino acid protein with a molecular weight of 50.5 kDa 5, 6. KRT80 is involved in cell differentiation, and is located near the desmosomal plaques during the early stage of differentiation, and dispersed throughout the cytoplasm in terminal differentiated cells. Keratin is an intermediate filament cytoskeletal protein that maintains the structural integrity of epithelial cells 7, and can be classified into epithelial and hair keratin. The expression profile of KRT80 suggests that it encodes a type II epithelial keratin, whereas the protein it encodes is more structurally similar to type II hair keratin 8. Keratin has tissue specificity, and is expressed in a differentiation-dependent manner 9. Keratin is widely used as a tumor marker in cancer diagnosis, and plays an active biological role in tumor cell proliferation and metastasis 10. Recently, abnormal KRT80 expression has been found in colorectal, gastric, and breast cancer, where it plays an important role in tumor development and progression 11-14. However, KRT80 expression in ovarian cancer, and its effect on malignant biological behavior, have not been reported.

ETS1 is a nuclear protein that mainly acts as a transcription activator, and is involved in the development of stem cells, cell senescence and apoptosis, and tumorigenesis 15. ETS1 contributes to tumor angiogenesis and invasion and migration of cancer cells, and promotes epithelial-mesenchymal transition (EMT) and the development of drug resistance. It is closely related to the occurrence, development, metastasis, and prognosis of tumors, and is overexpressed in many types of tumors 16, 17. The microRNA (miRNA) miR-206 is located on human chromosome 6p12.2, and impacts post-transcriptional regulation of gene expression in multicellular organisms by affecting mRNA stability and translation 18. Because it is downregulated in a variety of tumors, miR-206 is assumed to be a tumor suppressor 19, and also affects tumor cell proliferation, differentiation, invasion, metastasis, and other processes by regulating genes related to cell cycle, division, and apoptosis. For these reasons, miR-206 expression could be a biomarker of disease status and prognosis, and a predictor of drug resistance 20, 21.

In the present study, we examined the expression of KRT80 in ovarian tissue, and studied the relationship between KRT80 expression and clinicopathologic parameters and prognosis of ovarian cancer. We also studied the effect of KRT80 overexpression and knockdown on the malignant biological behavior of ovarian cancer cells *in vitro*. We further explored the relationship between the expression of KRT80 and the occurrence and development of ovarian cancer, revealing its regulatory pathways, transcription factors, and upstream miRNAs. These findings provide a new theoretical basis for further study of the mechanisms by which KRT80 participates in the occurrence and development of ovarian cancer. KRT80 may have applications as a diagnostic and prognostic indicator for ovarian cancer, and may represent a therapeutic target for novel targeted treatment regimens.

## Methods

### Source of specimen and clinical data

A total of 147 paraffin-embedded pathological specimens were collected from 2008 to 2014 at the Department of Obstetrics and Gynecology, Shengjing Hospital, China Medical University. According to the ethical and legal standards, all selected patients have obtained written informed consent. The study was approved by the Ethics Review Committee of Shengjing Hospital, China Medical University. All the ovarian malignant tumor samples were primary epithelial ovarian tumors. Normal ovarian tissues were from postmenopausal patients or from cervical cancer patients with double adnexal hysterectomy. All the cases have complete clinical data which can be obtained for each patient. Samples from patients who had received radiation therapy, chemotherapeutic, and hormone one therapy were excluded from the cohort. The pathological diagnosis of all tissue sections was performed by a Sheng Jing Hospital pathologist, and this sample set included 102 cases of ovarian epithelial malignant tumors (ovarian cancer group), 16 cases of ovarian epithelial borderline tumors (ovarian borderline group), 14 cases of benign tumors (ovarian benign group), and 15 cases of normal ovarian tissue (ovarian normal group). The mean ages of patients in each group were: 53.2 years in the ovarian cancer group (range: 16-79 years), 49.5 years in the borderline group (range: 19-84 years), 48.14 years in the benign group (range: 16-78 years), and 47.8 years in the normal group (range: 32-76 years). There was no significant difference in age between the groups (*P* > 0.05). In the ovarian cancer group, there were 67 cases of serous cystadenocarcinoma, 8 cases of mucinous adenocarcinoma, 19 cases of endometrioid adenocarcinoma, and 8 cases of clear cell carcinoma. According to histological classification, 27 cases were highly differentiated, 25 cases were moderately differentiated, and 50 cases had low differentiation. According to the International Federation of Obstetrics and Gynecology (FIGO, 2009), there were 24 cases in stage I, 19 cases in Stage II, 54 cases in stage III, and five cases in stage IV. There were 22 and 65 cases with and without lymph node metastasis, respectively, and 15 cases without lymph node dissection.

### Immunohistochemistry

Paraffin-embedded tissue samples from each group were fixed in 10% formalin and sectioned continuously at a thickness of 5 μm after conventional paraffin embedding. KRT80 expression was detected using an ultra-sensitive TM SP (mouse/rabbit) IHC kit (Maixin, China, Cat# KIT-9720). Rabbit KRT80 polyclonal antibody (Proteintech, Wuhan, China, Cat# 16835-1-AP) was used at a 1:500 dilution. Brown and yellow granules were observed in the cytoplasm, and were considered to be positive staining. A staining intensity score was adopted, with 0 indicating no coloring, 1 indicating light yellow, 2 indicating brown yellow, and 3 indicating brown. The percentage of stained cells in a visual field was scored according to the following: 0 (< 5%), 1 (5 ~ 25%), 2 (26 ~ 50%), 3 (51 ~ 75%), and 4 (> 75%). The final score was calculated as the product of the staining score and percentage of stained cells in a visual field, and was denoted as follows: 0 ~ 2 (-), 3 ~ 4 (+), 5 ~ 8 (+ +), and 9 ~ 12 (+ + +). Based on these scores, 0 ~ 4 was designated as the 'low expression group' and 5 ~ 12 as the 'high expression group'. Each sample was scored by two independent observers, and disagreements were resolved by a third independent observer.

### Cell culture

Ovarian cancer cell lines (CAOV3 and OVCAR3) and normal ovary epithelial cells (HOSEpiC) were purchased from the Cell Bank of the Chinese Academy of Sciences (Shanghai Institute of Biochemistry and Cell Biology, Shanghai, China). CAOV3, OVCAR3 and HOSEpiC cells were cultured in normal RPMI1640 medium (BI, USA) containing 10% fetal bovine serum (FBS). The cells were cultured at 37 °C under 5% CO_2_ and saturated humidity.

### Cell transfection and construction of stable transfected cell lines

Cells in the logarithmic growth phase were inoculated into 6-well plates the day before transfection. KRT80 small interfering RNA (siRNA) was constructed by GenePharma (Shanghai, China) to knock down KRT80. The KRT80 siRNA and negative control siRNA were transfected into CAOV3 and OVCAR3 cells, using lipofectamine (Lipofectamine 3000 transfection kit, GIBCO, Invitrogen). The sequence of KRT80 siRNA is: sense: 5'-GCUCCUGCGUGGUUGGCUUTT-3', antisense: 5'-AAGCCAACCACGCAGGAGCTT-3'. The sequence of its negative control is: sense: 5'-UUCUCCGAACGUGUCACGUTT-3', antisense: 5'-ACGUGACACGUUCGGAGAATT-3'. After transfection for 48 h, the cells were collected for analysis via real-time quantitative PCR (RT-qPCR), western blotting, and biological assays. The KRT80 overexpressing cell line and associated negative control cell line were constructed by transfecting CAOV3 and OVCAR3 cells using the GeneChem lentivirus gene transfection system. Stable cell lines were screened with 2 μg/ml puromycin (Solarbio, Beijing, China).

### RT-qPCR

Total RNA was extracted from transfected cells using the TRIzol reagent (Takara Bio, Inc., Shiga, Japan) according to the manufacturer's instructions. The purity and concentration of RNA were determined by ultraviolet spectrophotometer. RNA was reverse transcribed into cDNA using the Takara 047A kit (Takara Bio, Inc., Shiga, Japan), and RT-qPCR was performed using a 7500 Fast real-time PCR system. The amplification conditions were: denaturation at 95 °C for 30 s, 95 °C for 5 s, and 60 °C for 30 s, for a total of 40 cycles. KRT80 primer sequence: Forward: 5'-AACCAGGAGAAGGAGGAGATGAAGG-3', Reverse: 5'-CCAGTTCAGTCCGATGAAGACACTC-3'. GAPDH primer sequence: Forward: 5'-ACAACTTTGGTATCGTGGAAGG-3', Reverse: 5'-GCCATCACGCCACAGTTTC-3'. The abundance of KRT80 mRNA was determined using the comparative -ΔCt method.

### Western blotting

Cells were lysed via ultrasonication in RIPA cell lysis buffer at 4 °C for 30 min. The cell debris was pelleted via centrifugation at 4 °C for 30 min at 12000 rpm, and the supernatant containing the total protein was collected into a new Eppendorf tube. The total protein concentration was determined using a BCA assay. Loading buffer was added to each sample and the protein was denatured at 100 °C for 5 min. The protein samples were separated electrophoretically using 10% sodium dodecyl sulphate-polyacrylamide gel electrophoresis, and then transferred onto a polyvinylidene fluoride membrane for western blotting. The membrane was subsequently blocked for 2 h using 5% milk or bovine serum albumin, and incubated overnight at 4 °C with primary antibodies in blocking solution. The primary antibodies we used are as follows: KRT80 (Proteintech, rabbit polyclonal antibody, 1:1000), BCL2 (Proteintech, rabbit polyclonal antibody, 1:2000), BAX (Affinity, rabbit polyclonal antibody, 1:1000), CyclinD1 (CST, rabbit polyclonal antibody, 1:1000), MMP2 (Proteintech, rabbit polyclonal antibody, 1:1000), MMP9 (Proteintech, rabbit polyclonal antibody, 1:500), E-Cadherin (Proteintech, rabbit polyclonal antibody, 1: 2000), N-Cadherin (Proteintech, rabbit polyclonal antibody, 1:2000), MEK (Affinity, rabbit polyclonal antibody, 1:1000), p-MEK (Affinity, rabbit polyclonal antibody, 1:1000), ERK (CST, rabbit polyclonal antibody, 1:1000), p-ERK (CST, rabbit polyclonal antibody, 1:1000), ETS1 (CST, rabbit polyclonal antibody, 1:1000), GAPDH (ZSGB-BIO, mouse monoclonal antibody, 1:2000). The next day, the membrane was washed 3 times with 1×TBST for 10 min each. HRP-labeled goat anti-rabbit secondary antibody or murine IgG (1:2000, Zhongshan Jinqiao, China) was added to the membranes and incubated for 2 h, and then washed with 1×TBST 3 times for 10 min each. Protein expression was determined using a chemiluminescent HRP substrate (Millipore, Billerica, MA, USA).

### Cell proliferation test

Adhered cells were resuspended by tryptic digestion, and then diluted to 2×10^4^ cells/ml and inoculated into 96-well plates at 2000 cells/well. The initial time point, 0 h, was defined as the time when the suspended cells adhered to the wells. To measure cell proliferation, 10 μl of CCK8 reagent (Vazyme Biotech, Nanjing, China) was added to each well and incubated for 0, 24, 48, 72, and 96 h at 37 °C for 4 h. The optical density at 450 nm was measured at each time point.

### Cell cycle

Cells were removed from plates by digesting with trypsin without ethylenediaminetetraacetic acid (EDTA), and cell suspensions were collected by centrifugation at 1000 rpm for 5 min. The supernatant was removed, and 500 μl of pre-cooled 70% ethanol was added to fix the cells overnight at 4 °C. The next day the ethanol was removed by centrifugation, and the cells were washed with PBS. Thereafter, 500 μl of 9:1 PI/RNaseA staining solution (KeyGen Bio-tech, Nanjing, China) was added to each sample. The color was developed by incubating cells at room temperature for 30 min. The proportion of cells in the G0/G1, S, or G2/M phase was measured using flow cytometry on a BD FACSDiva system (BD Biosciences, New York, USA).

### Scratch test

Cell suspensions were prepared from logarithmic growth phase cells and inoculated into 6-well plates. When the cells grew to 90% confluency, the tip of a 200 μl pipette tip was used to gently scratch the surface of the 6-well plates. After washing 2~3 times with PBS, the cell fragments were removed and cultured in serum-free medium. The scratch width was observed and measured at 0 h and 24 h under a microscope.

### Transwell assay

Matrigel (BD Biosciences, USA) and serum-free medium were mixed at a ratio of 1:7.5, and 70 μl was added to the upper chambers of each transwell chamber (Corning Coster) and placed in an incubator at 37 °C overnight. Thereafter, 500 μl of RPMI1640 medium containing 20% FBS was added to the lower chamber, and 200 µl of serum-free cell suspension (2×10^5^ cells/ml) was added to the upper chamber. The cells were incubated at 37 °C under 5% CO_2_ for 24 or 48 h. Thereafter, cells were washed with PBS and fixed in 4% paraformaldehyde for 30 min, washed again with PBS, and stained with 0.1% crystal violet for 30 min. The upper chamber surface was lightly swabbed with a cotton swab, and the number of ovarian cancer cells infiltrating the chamber was counted under a microscope.

### Chromatin immunoprecipitation (ChIP)

Ovarian cancer cells in the logarithmic phase were collected. The ChIP assay was performed using the Simple ChIP Enzymatic Chromatin IP Kit (#9004, Cell Signaling Technology, California, USA) according to the manufacturer's instructions. Cell samples were processed by preparation of the nuclei, digestion and immunoprecipitation the chromatin, immune complex precipitation and washing, elution of the chromatin, and reversal of cross-linking and DNA purification. ETS1 was visualized by the addition of 10 μl of ETS1 antibody (CST, [rabbit], 14069S) and 2 μg of IgG antibody (CST, [mouse], 5415S). DNA from the immunoprecipitation was subsequently amplified via RT-qPCR using the specific primer. The binding site primer sequence: Forward: 5'-TGACCGTCTATGTCCTCC-3', Reverse: 5'-CTCCCTGGCTTATCTTCC-3'.

### Dual luciferase reporter gene assay

The plasmid for the double luciferase reporter gene assay was synthesized by GenePharma (Shanghai, China). A wild-type (WT) fragment (ETS1-WT), and corresponding mutant (Mut) fragment (ETS1-MUT), containing the miR-206 potential binding site were inserted into the reporter vector to construct the ETS1 luciferase reporter vector. HEK293T cells were cultured on a 24-well plate and co-transfected with miR-206 mimic or miR-Mock and WT or Mut report vector when 70% of HEK293T cells were fused. After transfection for 48 hours, whole cell lysate was collected and luciferase activity was measured by luciferase reporter assay (Promega, Madison, WI, USA), according to the manufacturer's instructions.

### Bioinformatics

The Oncomine database (http://www.oncomine.org) was used to analyze the expression of KRT80 mRNA in different types of cancer. GEPIA (http://gepia.cancer-pku.cn/) was used to analyze the differential expression of KRT80 in ovarian cancer and normal ovarian tissue. The changes in KRT80 expression in patients with ovarian cancer were assessed using the cBioPortal repository (http://www.cbioportal.org), and the co-expressed genes were screened. The prognostic utility of KRT80 in ovarian cancer was studied using Kaplan-Meier survival plots (http://kmplot.com). Functional and pathway enrichment analysis of KRT80-related genes was performed using the David database (https://david.ncifcrf.gov), which provides a systematic and comprehensive biological functional annotation. The C2.cp.kegg.v6.2.symbols.gmt dataset was downloaded from the MsigDB database on the gene set enrichment analysis (GSEA) website, and the GSEA 3.0 software was used for genome enrichment analysis. The Promo database (http://alggen.lsi.upc.es/cgibin/promo_v3/promo/promoinit.cgi?dirDB=TF_8.3) and JASPAR database (http://jaspar.genereg.net/) were then used to predict the transcription factor/s of KRT80, and the StarBase database (http://starbase.sysu.edu.cn/) was used to analyze the correlation between ETS1 mRNA and KRT80 mRNA. The TargetScan database (http://www.targetscan.org/) and miRDB database (http://mirdb.org/) were used to predict miRNAs upstream of ETS1, and possible binding sites.

### Statistical analysis

The data were analyzed using SPSS 21.0 (IBM Corporation, Armonk, NY, USA), and graphs was generated in GraphPad Prism 8.0. All data are represented as the mean±SD. T-tests and chi-squared tests were used to compare the differences between two groups. One-way analysis of variance (ANOVA) was used for comparison between two or more groups, and Kaplan-Meier and log-rank tests were used for survival curve analysis. Univariate and multivariate Cox regression models were used to analyze the risk factors that affect prognosis. A bilateral *P* < 0.05 was considered to indicate statistical significance. * * *, *P* < 0.001; * *, *P* < 0.01; *, *P* < 0.05.

## Results

### Expression and clinical significance of KRT80 in different ovarian tissues

**Figure 1:**
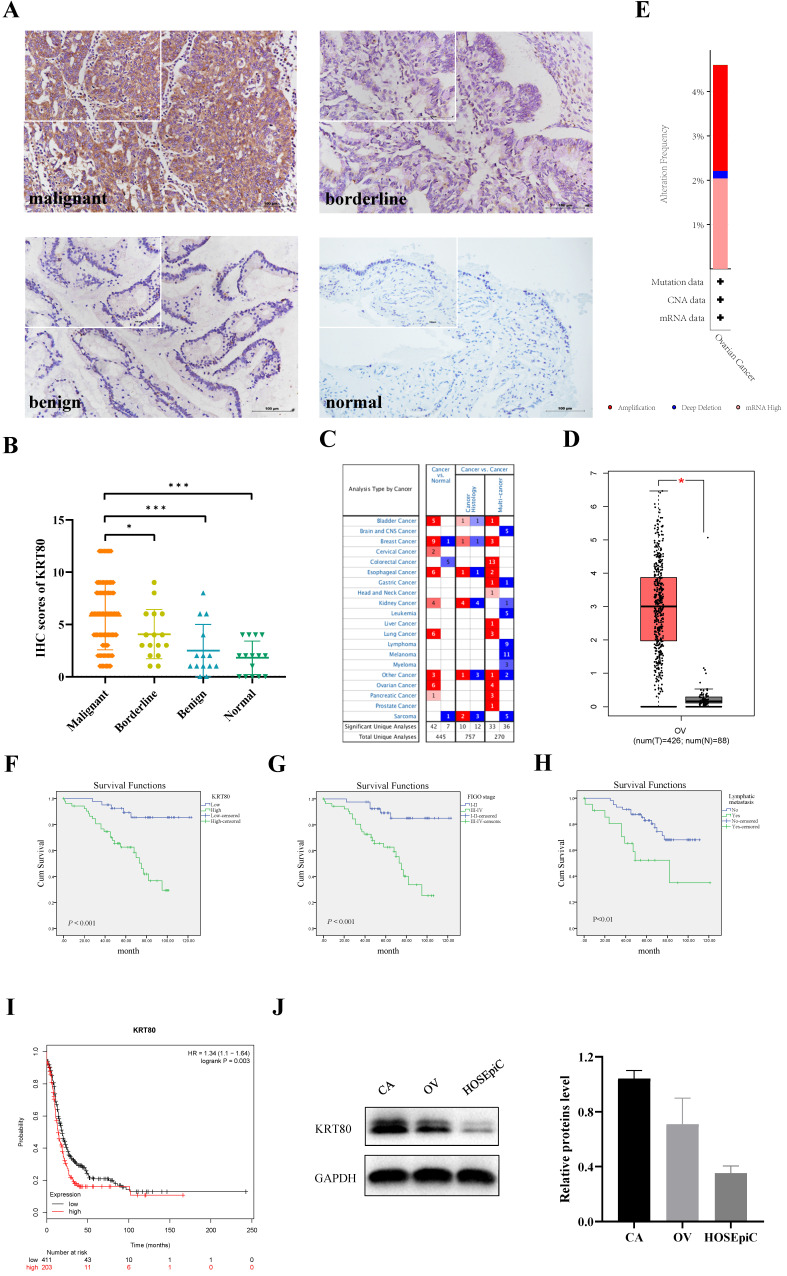
** High KRT80 expression in patients with ovarian cancer associated with poor prognosis. (A)** KRT80 expression in ovarian tissue samples (×200, scale bar = 100 µm; upper left ×400): ovarian malignant tumor (n = 102), ovarian borderline tumor (n = 16), ovarian benign tumor (n = 14), ovarian normal tissue (n = 15). **(B)** Immunostaining scores of KRT80 in malignant, borderline, benign, and normal ovarian tissues. **(C)** KRT80 mRNA expression in the various tumors from Oncomine database. The cell numbers indicate the number of analyses that meet the thresholds. The color intensity (red or blue) is directly proportional to the significance level of upregulation or downregulation. Table header was divided into Cancer vs. Normal and Cancer vs. Cancer, indicating the differential expression of KRT80 in cancer and normal tissues and in different cancer tissues, respectively. **(D)** KRT80 mRNA expression in the GEPIA database. Box plots show KRT80 mRNA expression in ovarian tumor (red plot) and the corresponding normal tissues (gray plot). Axis unit is log_2_(TPM + 1). **(E)** KRT80 genetic variation analysis in cBioPortal **(F,G,H)**. Overall survival analysis according to KRT80 expression, FIGO stage and lymphnode metastasis. **(I)** KRT80 expression with PFS in Kaplan-Meier Plotter. **(J)** Representative images and quantitation of the western blotting showed that the protein expression of KRT80 in the ovarian cancer cell lines (CAOV3 and OVCAR3) and normal ovary epithelial cells (HOSEpiC) (n = 3). GAPDH was used as an internal control. Data are presented as mean ± SD.

KRT80 protein was primarily expressed in the cytoplasm (Figure 1A), and was expressed in ovarian tissue in all groups. KRT80 expression increased gradually with the progression of malignant ovarian cancer. The positive expression rate of KRT80 in the ovarian cancer group (79.41%, 81/102) was significantly higher than that in the normal (26.67%, 4/15) and benign (28.57%, 4/14) groups (*P* < 0.001). The positive expression rate of KRT80 in the ovarian cancer group (79.41%, 81/102) was also higher than that in ovarian borderline group (75.00%, 12/16), but the difference was not statistically significant (*P* > 0.05). We used a scoring metric to define the expression of KRT80, with (++) and (+++) denoting high expression. The high expression rate of KRT80 in the ovarian cancer group (56.86%, 58/102) was significantly higher than that in the normal (0) and benign (7.14%, 1/14) groups (*P* < 0.001). The high expression rate of KRT80 in the ovarian cancer group (56.86%, 58/102) was also higher than that in the ovarian borderline group (31.25%, 5/16), although this was not statistically significant (*P* > 0.05). The positive expression rate of KRT80 in the ovarian borderline group (75.00%, 12/16) was higher than that in the normal (26.67%, 4/15) and benign (28.57%, 4/14) groups (*P* < 0.05). The high expression rate of KRT80 in the borderline tumor group (31.25%, 5/16) was also higher than that in the normal (0) (*P* < 0.05) and benign (7.14%, 1/14) (*P* > 0.05) groups (Table 1) (Figure 1B). According to the data in the Oncomine database, KRT80 is overexpressed in many different cancer tissues (Figure 1C). In the GEPIA database, the expression of KRT80 in 426 ovarian cancer patients was significantly higher than that in 88 normal ovarian tissue samples (*P* < 0.05) (Figure 1D). In the cBioPortal database, 587 cases of ovarian cancer with KRT80 gene changes were analyzed, and the mutation rate of KRT80 was 4.6%, including 2.39% amplification, 0.17% deep deletion, and 2.04% high mRNA in TCGA (Firehose Legacy) (Figure 1E).

A total of 102 ovarian cancer specimens were evaluated in the present study. The relationship between the expression of KRT80 and clinicopathologic parameters is shown in Table 2. A high expression rate of KRT80 was significantly correlated with FIGO stage and lymph node metastasis (*P* < 0.01). The high expression rate of KRT80 was 69.49% in the late FIGO group (stage III ~ IV), which was higher than that in the early FIGO group (stage I ~ II) (39.53%). The positive rate of KRT80 in the lymph node metastasis group (81.82%) was higher than that in non-metastasis group (44.62%). However, KRT80 expression was not correlated with age, histological grade, or pathological type (*P* > 0.05).

A total of 102 patients with ovarian cancer were followed up until August 30, 2020. In Kaplan-Meier survival analysis, 102 ovarian cancer patients were divided into low KRT80 expression group (44 cases) and high KRT80 expression group (58 cases); FIGO I-II stage group (43 cases) and FIGO III-IV stage group (59 cases); no lymphatic metastasis group (65 cases) and lymphatic metastasis group (22 cases). The 5 year survival rate of patients with high KRT80 expression was significantly lower than that of patients with low KRT80 expression (*P* < 0.001) (Figure 1F). FIGO stage (*P* < 0.001) (Figure 1G) and lymph node metastasis (*P* < 0.01) (Figure 1H) were significantly correlated with the overall survival, but the age, histological grade, and pathological type were not (*P* > 0.05). In the KM-plot database, patients with high KRT80 expression had significantly shorter progression-free survival than those with low expression of KRT80 (*P* < 0.01) (Figure 1I).

The relationship between different clinicopathological parameters and prognosis was evaluated by Cox regression analysis. Single-factor Cox regression analysis showed that the expression of KRT80, FIGO stage, and lymph node metastasis were risk factors for the prognosis of ovarian cancer (*P* < 0.01). Multivariate Cox regression analysis showed that KRT80 expression and FIGO stage were independent risk factors for prognosis (*P* < 0.05) (Table 3).

### KRT80 promotes ovarian cancer by inducing cell proliferation and cell cycle progression

The expression of KRT80 protein in ovarian cancer cell lines and normal ovary epithelial cells was detected by western blotting. The results demonstrate that the protein expression level of KRT80 in CAOV3 and OVCAR3 was higher than that in HOSEpiC. Moreover, the expression of KRT80 was highest in CAOV3 cells (Figure 1J).

**Figure 2:**
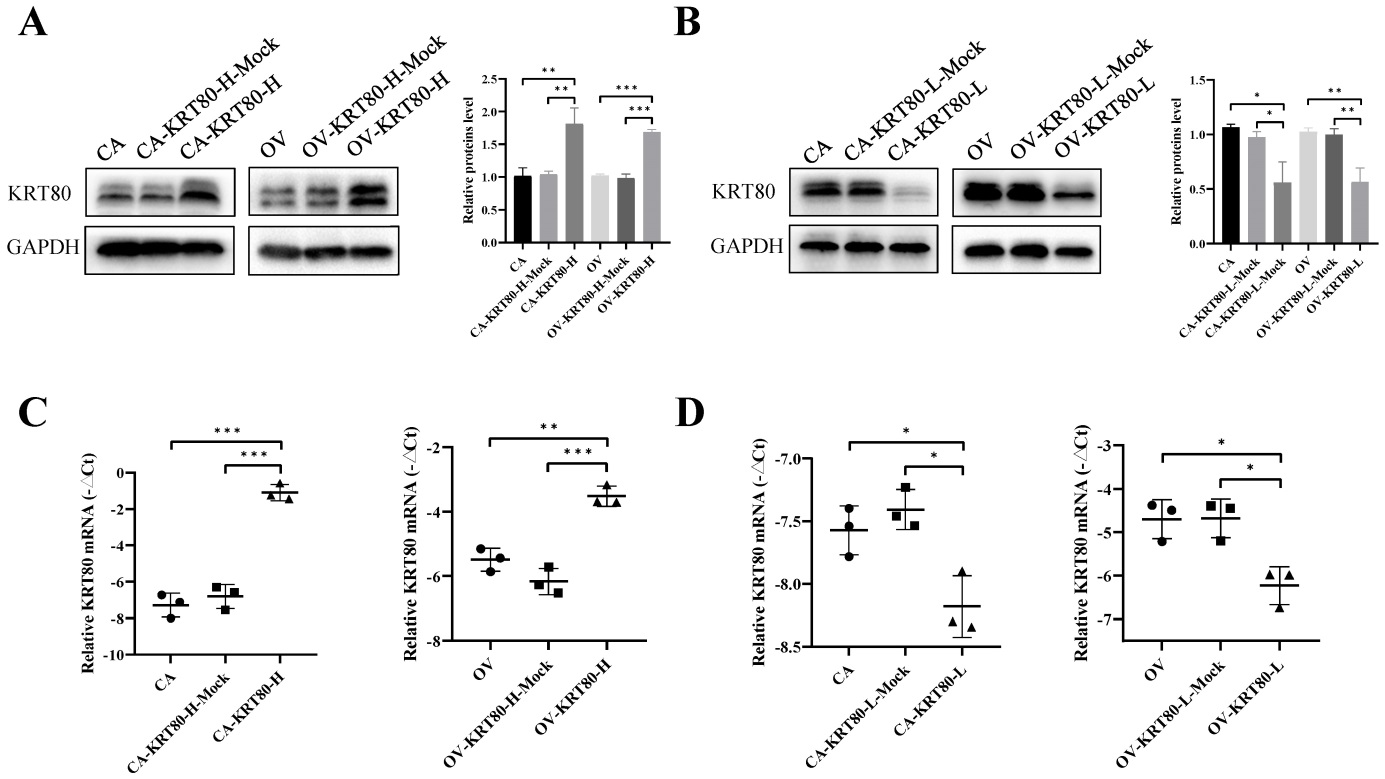
**The protein and mRNA expression of KRT80 in the KRT80 overexpression/knockdown groups. (A,B)** Representative images and quantitation of the western blotting showed that the protein expression of KRT80 in the KRT80 overexpression/knockdown groups (n = 3). GAPDH was used as an internal control. **(C,D)** The relative KRT80 mRNA expression in the KRT80 overexpression/knockdown groups (n = 3). Data are presented as mean ± SD. *, *P* < 0.05; **, *P* < 0.01; ***, *P* < 0.001.

Overexpression cell lines of KRT80 (CAOV3-KRT80-H and OVCAR3-KRT80-H) and knockdown cell lines of KRT80 (CAOV3-KRT80-L and OVCAR3-KRT80-L) were constructed using CAOV3 and OVCAR3 cells. Changes in KRT80 expression at the mRNA and protein levels were confirmed using qRT-PCR and western blotting, respectively (Figure 2A-D).

**Figure 3:**
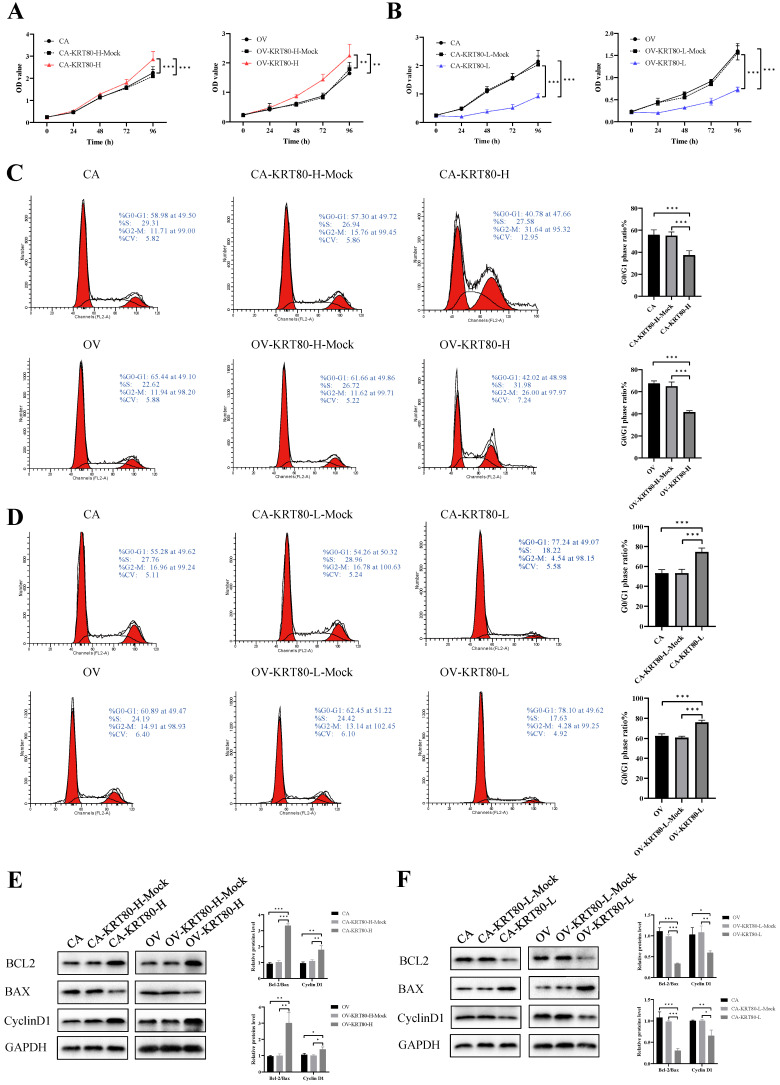
**The influences of KRT80 on proliferation and cell cycle in ovarian cancer cells. (A)** Overexpression of KRT80 promoted cell proliferation of ovarian cancer cells in CCK8 assay (n = 9). **(B)** Knockdown of KRT80 inhibited cell proliferation of ovarian cancer cells in CCK8 assay (n = 9). **(C)** Overexpression of KRT80 promoted ovarian cancer cell cycle passed into S and G2/M phases (n = 6). **(D)** Knockdown of KRT80 duced ovarian cancer cell cycle arrest in the G0/G1 phase (n = 6). **(E,F)** Representative images and quantitation of the western blotting showed that the protein expression of BCL2, BAX and CyclinD1 in the KRT80 overexpression/knockdown groups (n = 3). GAPDH was used as an internal control. Data are presented as mean ± SD. *, *P* < 0.05; **, *P* < 0.01; ***, *P* < 0.001.

The progression of these cell lines was evaluated by measuring cell proliferation rates and cell cycle arrest after KRT80 overexpression/knockdown. The CCK8 assay showed that KRT80 overexpression could significantly increase the proliferation rate of cells (*P* < 0.05) (Figure 3A). In contrast, the cell proliferation rate after KRT80 knockdown was significantly lower than that in the control group (*P* < 0.05) (Figure 3B). KRT80 overexpression promoted changes in the cell cycle from G1 phase to S phase (*P* < 0.05) (Figure 3C). Conversely, KRT80 knockdown induced cell cycle arrest in the G0/G1 phase (*P* < 0.05) (Figure 3D). The overexpression of KRT80 increased the expression of BCL2 and CyclinD1, but decreased the expression of BAX (Figure 3E). In contrast, after KRT80 knockdown, the expression of BCL2 and CyclinD1 decreased and the expression of BAX increased (Figure 3F). These results suggest that high KRT80 expression promotes the proliferation of ovarian cancer cells, and the transition of ovarian cancer cells from G1 phase to S phase.

### KRT80 promotes invasion and migration of ovarian cancer cells

**Figure 4:**
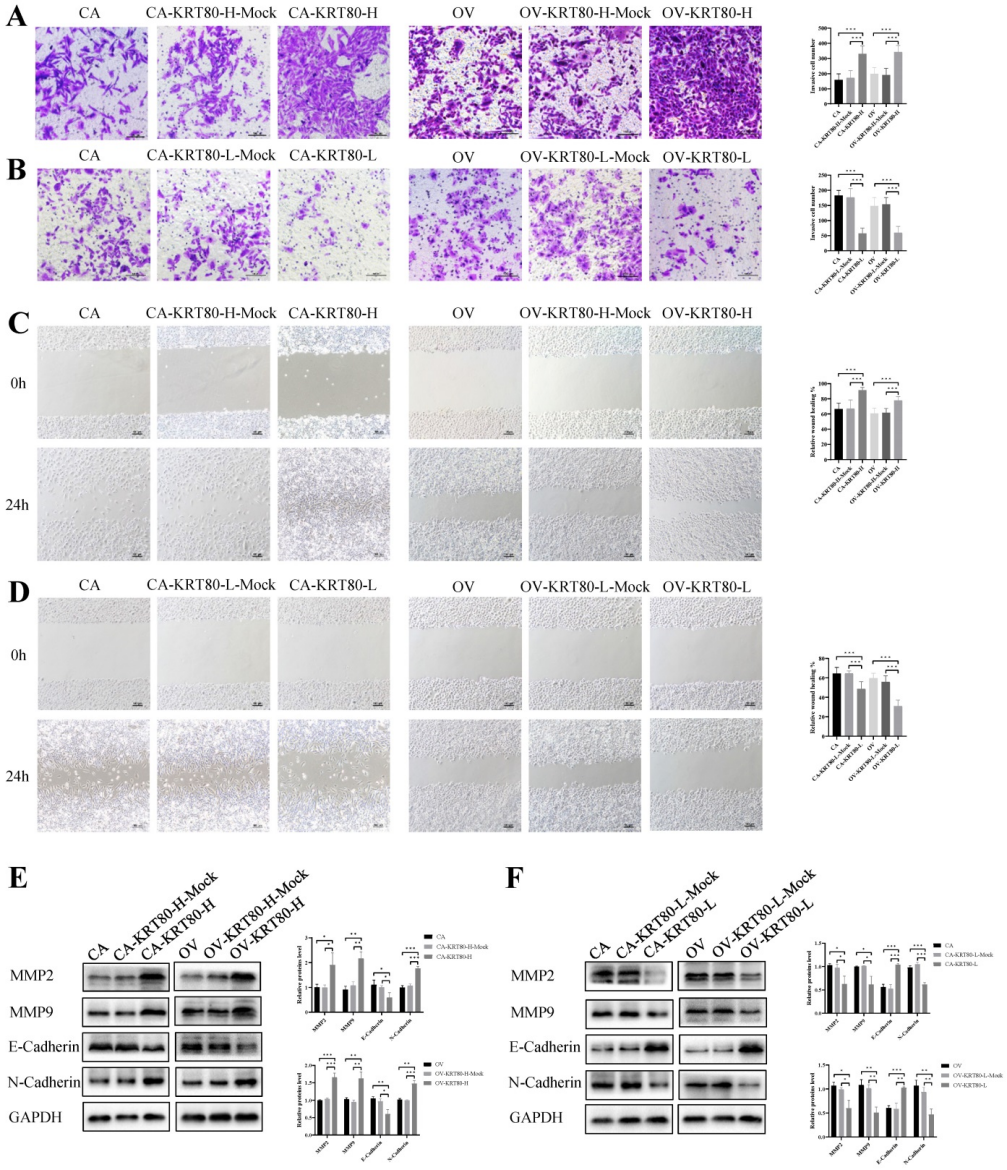
**KRT80 promoted invasion and migration in ovarian cancer cells. (A)** Overexpression of KRT80 promoted ovarian cancer cells invasion (n = 9; ×200, scale bar = 100 µm). **(B)** Knockdown of KRT80 suppressed ovarian cancer cells invasion (n = 9; ×200, scale bar = 100 µm). **(C)** Overexpression of KRT80 promoted ovarian cancer cells migration (n = 9; ×100, scale bar = 100 µm). **(D)** Knockdown of KRT80 inhibited ovarian cancer cells migration (n = 9; ×100, scale bar = 100 µm). **(E,F)** Representative images and quantitation of the western blotting showed that the protein expression of MMP2, MMP9, E-cadherin and N-cadherin in the KRT80 overexpression/knockdown groups (n = 3). GAPDH was used as an internal control. Data are presented as mean ± SD. *, *P* < 0.05; **, *P* < 0.01; ***, *P* < 0.001.

The effects of overexpression/knockdown of KRT80 on cell invasion and migration capabilities were evaluated. KRT80 overexpression could significantly enhance the invasive ability of cells (*P* < 0.05) (Figure 4A), whereas KRT80 knockdown decreased the invasive ability of cells (*P* < 0.05) (Figure 4B). After KRT80 overexpression, the wound healing speed of the cells was faster than that of the control group, and cell migration was enhanced (*P* < 0.05) (Figure 4C). After KRT80 knockdown, the wound healing speed was slower than that of the control group, and cell migration decreased (*P* < 0.05) (Figure 4D). With KRT80 overexpression, MMP2, MMP9, and N-cadherin expression were upregulated, whereas E-cadherin expression was downregulated (*P* < 0.05) (Figure 4E). The reverse effect was observed with KRT80 knockdown (*P* < 0.05) (Figure 4F). These results further confirmed that KRT80 promotes the invasion, migration, and EMT of ovarian cancer cells.

### KRT80 activates the MEK/ERK signaling pathway

**Figure 5:**
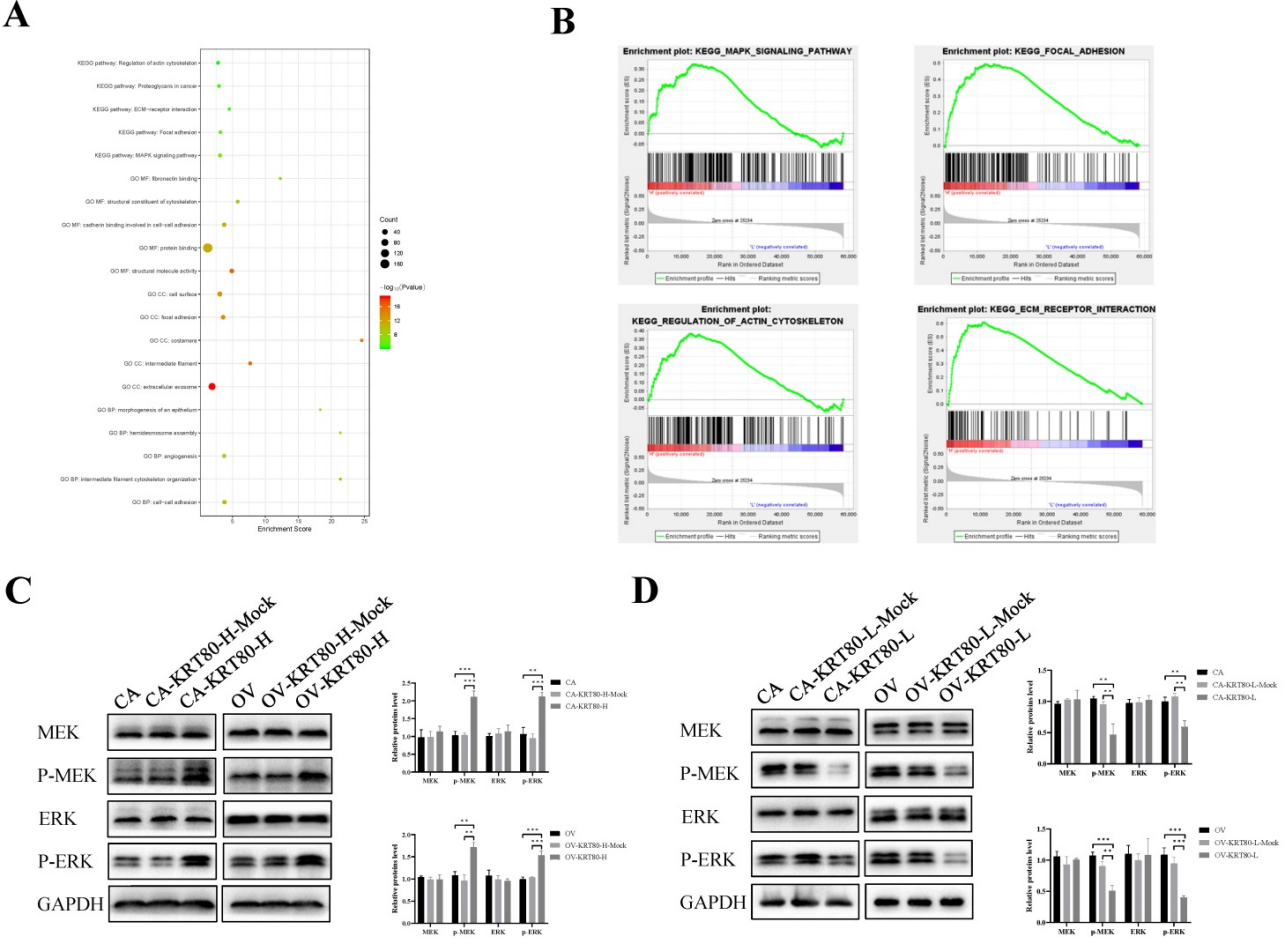
** KRT80 activated the MEK/ERK signaling pathway. (A)** The bubble plot of top 20 biological functions and pathways of coexpressed genes of KRT80. **(B)** GSEA analysis of KRT80-related enrichment gene sets. KEGG_MAPK_SIGNALING_PATHWAY (NES = 1.72, *P*-value = 0.022, FDR = 0.083); KEGG_FOCAL_ADHESION (NES = 2.28, *P*-value = 0.002, FDR = 0.006); KEGG_REGULATION_OF_ACTIN_CYTOSKELETON (NES = 2.08, *P*-value = 0.000, FDR = 0.022); KEGG_ECM_RECEPTOR_INTERACTION (NES = 2.10, *P*-value = 0.002, FDR = 0.022). **(C,D)** Representative images and quantitation of the western blotting showed that the protein expression of MEK, ERK, p-MEK and p-ERK in the KRT80 overexpression/knockdown groups (n = 3). GAPDH was used as an internal control. Data are presented as mean ± SD. *, *P* < 0.05; **, *P* < 0.01; ***, *P* < 0.001.

We used the cBioPortal to screen genes that are co-expressed with KRT80, and then used the David database to perform GO enrichment analysis on the KRT80-related genes. A bubble chart containing the top 20 GO terms (BP/CC/MF) and KEGG pathways related to KRT80 was generated in R using the package ggplot2 (Figure 5A). In order to further explore the molecular mechanism and biological function of KRT80 in ovarian cancer, we analyzed it. GO analysis of biological processes showed that KRT80-related genes were mainly involved in cell-cell adhesion, intermediate filament cytoskeleton organization and angiogenesis. Cellular component analysis indicated that KRT80-related genes were abundant in the extracellular exosome, intermediate filament and focal adhesion. Molecular function analysis indicated that KRT80-related genes were mainly involved in the function of protein binding, structural molecule activity and structural constituent of cytoskeleton. Furthermore, KEGG analysis showed that KRT80-related genes were enriched in MAPK signaling pathway, focal adhesion, ECM-receptor interaction, proteoglycans in cancer and regulation of actin cytoskeleton signaling pathways. Additionally, further pathway enrichment analysis in the GSEA database has been carried out and consistent results have been obtained. Pathway enrichment analysis suggested that KRT80 was related to the MAPK signaling pathway, focal adhesion, actin cytoskeleton regulation, and ECM-receptor interactions (Figure 5B). These signaling pathways are related to core biological carcinogenic processes. Regulation of focal adhesion and reorganization of actin cytoskeleton are crucial determinants of cell migration. Moreover, ERK/MAPK signaling plays an important role during actin and adhesion modulation which is associated with tumor cell invasion 22. These findings suggest that KRT80 may play an important role in the development of ovarian cancer.

The effect of KRT80 on the MAPK signaling pathway was confirmed via western blotting. KRT80 overexpression increased the expression of p-MEK and p-ERK (*P* < 0.05), but the protein levels of MEK and ERK did not change significantly (*P* > 0.05) (Figure 5C). Similarly, the expression of p-MEK and p-ERK decreased with KRT80 knockdown (*P* < 0.05), but the protein expression levels of MEK and ERK did not change significantly (*P* > 0.05) (Figure 5D). This confirms that KRT80 activates the MEK/ERK signaling pathway.

### ETS1 regulates KRT80 expression at the transcriptional level

**Figure 6:**
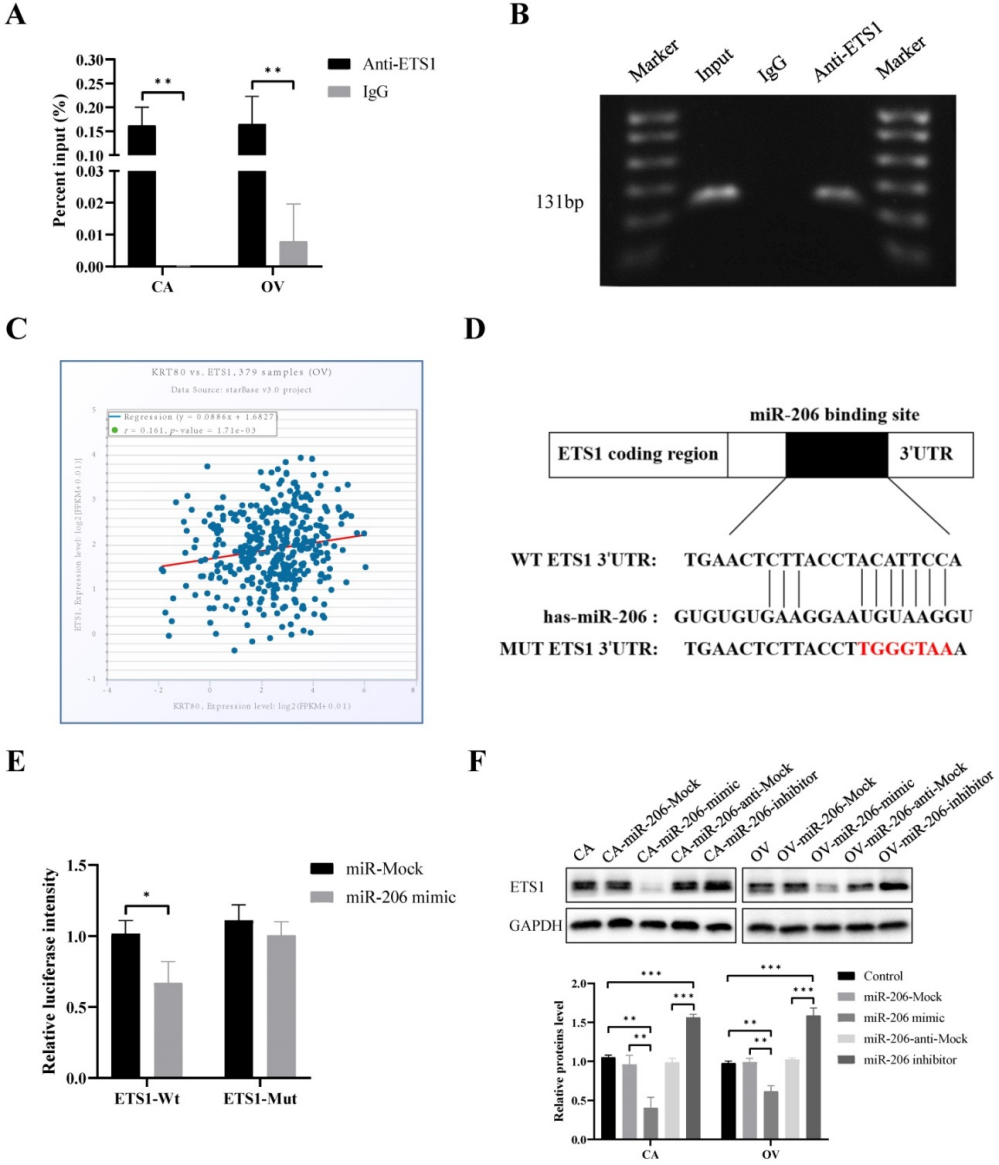
** Transcription factor ETS1 regulated KRT80 expression and ETS1 is a target of miR-206 in ovarian cancer cell. (A)** ChIP-PCR showed that ETS1 could bind the promoter region of KRT80 in ovarian cancer cells using ETS1 primary antibody, IgG was used as a negative control (n = 3). **(B)** ChIP-PCR products were analyzed using horizontal agarose gel electrophoresis and visualized using UV. **(C)** Correlation between KRT80 mRNA and ETS1 mRNA in 379 ovarian samples using starBase. **(D)** Binding site between miR-206 and 3ʹ-UTR of ETS1. **(E)** Dual Luciferase Gene Reporter System for detection of luciferase activity in ETS1-WT or ETS1-MUT and miR-206 mimic or miR-Mock co-transfection. **(F)** Representative images and quantitation of the western blotting showed that the protein expression of ETS1 in the miR-206 mimics/inhibitor groups (n = 3). GAPDH was used as an internal control. Data are presented as mean ± SD. *, *P* < 0.05; **, *P* < 0.01; ***, *P* < 0.001.

We predicted that ETS1 is the transcription factor that regulates KRT80 expression using the Promo and JASPAR databases. ChIP and microarray analysis were used to confirm that ETS1 bound to the promoter -616 bp to -486 bp sequence upstream of KRT80 (Figure 6A, B). ETS1 mRNA was positively correlated with KRT80 mRNA in 379 cases of ovarian cancer (*P* < 0.05, r = 0.161) (Figure 6C). These results suggest that ETS1 regulates KRT80 expression at the transcriptional level.

### ETS1 is the direct target of miR-206 in ovarian cancer cells

The TargetScan and miRDB databases together predicted that ETS1 was a potential target for miR-206, with potential binding sites located between the 3ʹ-UTR of ETS1 and miR-206 (Figure 6D). According to a dual luciferase reporter gene assay, the relative luciferase activity of the miR-206 mimic and wild-type ETS1 3ʹ-UTR (ETS1-WT) co-transfected group was significantly lower than that of the control group (*P* < 0.05). However, there was no significant difference in the luciferase activity between the miR-206 mimic and mutant ETS1 3ʹ-UTR (ETS1-MUT) co-transfected group and the control group (*P* > 0.05) (Figure 6E). The protein expression of ETS1 was inhibited after transfection with the miR-206 mimic (*P* < 0.05). After transfection with the miR-206 inhibitor, ETS1 expression increased (*P* < 0.05) (Figure 6F). The above results confirm that ETS1 is the direct target of miR-206 in ovarian cancer cells. In addition, our bioinformatics analysis of multiple databases showed that KRT80 was not a potential target for miR-206. Meanwhile, we detected by western blotting and founded that the protein expression level of KRT80 did not change significantly after transfection with the miR-206 mimic and miR-206 inhibitor (P > 0.05) (Figure S1). Therefore, we speculate that miR-206 has no direct effect on KRT80, but through ETS1 which is a direct target of miR-206 in ovarian cancer cells. And ETS1 further regulates KRT80 expression at the transcriptional level.

## Discussion

Ovarian cancer is the leading cause of death among gynecological malignant tumors. Because of the lack of typical early clinical symptoms and diagnostic methods, it is usually only discovered in the late stages. Intraperitoneal diffusion, extraperitoneal metastasis, and chemotherapy resistance all contribute to the low survival rate of ovarian cancer 3. Therefore, it is essential to explore the mechanisms by which ovarian cancer develops to enable early screening, prevention, and treatment.

Keratin is an intermediate filament cytoskeletal protein that plays an important role in maintaining the stability and integrity of epithelial cells. Keratin also participates in signal transduction processes, such as intracellular cell stress, proliferation, and metabolism 23, 24. Keratin is widely present in tumors, plays an important role in the functional regulation of cancer cells, and is a molecular marker of many types of tumors 25-27. KRT80 exists in almost all types of epithelium, and is a representative marker of epithelial cells. KRT80 expression is related to the advanced differentiation of tissue or cells 7, 9.

To date, there have been few studies on KRT80 expression in malignant tumors. KRT80 is overexpressed in colorectal, gastric, and breast cancers, and esophageal squamous cell carcinoma 11-14, 28, but has not been studied in ovarian cancer. In the present study, we identified the high expression of KRT80 in ovarian cancer tissue by immunohistochemical analysis, and further confirmed this result using cancer databases. The positive expression rate of KRT80 was related to FIGO stage and lymph node metastasis, but not to the age, histological grade, and pathological type of the patient. This could be expanded in other cancer types, given that KRT80 clearly plays a role in ovarian cancer and other cancer types. JL et al. 12 showed that KRT80 expression in colorectal cancer was significantly higher than that in para-cancerous normal tissue, and was significantly correlated with lymph node metastasis and pathological stage. In the present study, Kaplan-Meier analysis showed that KRT80 expression was correlated with the prognosis of ovarian cancer patients, which was consistent with the KM-plot database. KRT80 is an independent risk factor for the prognosis of patients with ovarian cancer. JM et al. 29 found that KRT80 participates in the construction of the miRNA-mRNA network in colorectal cancer exosomes, which is related to the staging, MSI phenotype, and prognosis of colorectal cancer, and plays an important role in the progression of colorectal cancer. Therefore, we concluded that KRT80 plays an important role in the development of ovarian cancer, and may be a prognostic indicator of ovarian cancer.

In the present study, overexpression and knockdown of KRT80 promoted the proliferation of ovarian cancer cells, the change from G0/G1 phase to S phase, and promoted the invasion, migration, and progression of EMT in ovarian cancer cells. These results suggest that KRT80 expression has a cancer-promoting effect in ovarian cancer. JL et al. 12 showed that inhibiting KRT80 expression significantly reduced the survival and proliferation of colorectal cancer cells, suggesting that KRT80 plays an important role in promoting the proliferation of colorectal cancer cells by regulating cell cycle and DNA replication. MW et al. 28 found that inhibition of KRT80 expression in esophageal squamous cell carcinoma inhibited the proliferation, invasion, and migration of esophageal squamous cell carcinoma (ESCC) cells. However, KJ et al. 30 found that the overexpression of TCONS_00049140 in mouse melanocytes downregulated the expression of KRT80, enhanced cell proliferation, and increased melanin production, indicating that KRT80 acts as a tumor suppressor gene in mouse melanoma. Taken together, KRT80 plays an important role in the development of various tumors, and should be studied further in ovarian cancer.

To further explore the mechanisms by which KRT80 affects the malignant biological behavior of ovarian cancer cells, we performed pathway enrichment analysis in the GSEA database and found that KRT80 is related to the MAPK signaling pathway. Mitogen-activated protein kinases (MAPK) are serine/threonine protein kinases that can be activated by different extracellular stimuli. The MAPK pathway plays an important role in cell proliferation, differentiation, apoptosis, angiogenesis, and tumor metastasis 31, 32. Keratins mediate tumor progression by activating the MEK/ERK signaling pathway. In hepatocellular carcinoma, hepatocyte growth factor activates the c-MET and MEK-ERK1/2 pathways, and upregulates the expression of KRT19. In addition, downstream transcription activators AP1 and SP1 of ERK1/2 activate the expression of KRT19 in hepatoma cells 33. Sunitinib reduced the expression of KRT6A by suppressing ERK1/2 and p38 MAPK signal transduction pathways in a skin model 34. KRT80 promotes tumor progression by activating the AKT signaling pathway. HS et al. 13 found that CIRCPIP5K1A interacts with miR-671-5p to further regulate the expression of KRT80, regulate the proliferation, invasion, and migration of gastric cancer cells, and play a role in carcinogenesis by activating the PI3K/Akt pathway. CL et al. 11 found that KRT80 could interact with DNA-activated catalytic polypeptide (PRKDC) via the AKT pathway to increase the migration and invasion of colorectal cancer cells. In the present study, we verified that the expression of MEK/ERK signaling pathway proteins were dysregulated in ovarian cancer cells with overexpression or knockdown of KRT80. This confirms that KRT80 promotes the malignant biological behavior of ovarian cancer cells by activating the MEK/ERK signaling pathway.

ETS1, a transcription factor with transcriptional activation, is involved in many biological functions, such as tumor cell proliferation, apoptosis, invasion, metastasis, and angiogenesis. At present, many studies have found that ETS1 expression is increased in colorectal, breast, prostate, gastric, and pancreatic cancers, and other malignant tumors 16, 35-38. A previous study also found that ETS1 is highly expressed in ovarian cancer, and is associated with a poor prognosis 39, 40. In ovarian cancer, VEGF induces ETS1 expression by activating the PI3K/Akt and p38MAPK signaling pathways, which further activates MMP9 and MMP13 and promotes SKOV3 invasion and metastasis 41. In addition, transcription factors are involved in the regulation of KRT80 expression during tumor progression. YP et al. 14 found that SREBP1 could target the enhancer of KRT80 and upregulate the expression of KRT80, which could promote cytoskeletal rearrangements at the leading edge, increase focal adhesion and cellular stiffening, and promote the invasion of breast cancer cells. In the present study, we demonstrated for the first time that ETS1 bound at the promoter located -616 bp to -486 bp upstream of KRT80 to regulate KRT80 expression. According to the StarBase database, ETS1 mRNA levels are positively correlated with KRT80 mRNA levels. Therefore, we hypothesize that ETS1 regulates KRT80 expression at the transcriptional level.

The miRNA miR-206 is a common tumor suppressor molecule, and its expression is decreased in lung cancer, breast cancer, renal cell carcinoma, endometrial cancer, and many other cancers 42-45. miRNA can bind to 3ʹ-untranslated regions (3ʹ-UTRs) and affect downstream target expression in cancer 46. MW et al. 28 found that miR-143-3p affects the malignant phenotype of ESCC cells by targeting KRT80 in esophageal squamous cell carcinoma. The expression level of miR-206 in ovarian cancer is lower than that in normal tissue. KIF2A mRNA contains two binding sites for miR-206, which inhibits the proliferation, migration, and invasion of ovarian cancer cells, and induces apoptosis 47. In the present study, a dual luciferase reporter assay showed that miR-206 could significantly inhibit the fluorescence intensity of the ETS1-Wt vector, but not the ETS1-Mut vector. This confirms that ETS1 was the direct target of miR-206 in ovarian cancer cells.

In the present study, we confirmed that KRT80 promotes ovarian cancer progression through the MEK/ERK pathway. Interestingly, miR-206 has the potential to promote tumorigenesis through the MEK/ERK pathway. YW et al. 48 found that CircTCF25 induces signal transduction in the MEK/ERK and AKT/mTOR pathways by inhibiting miR-206 expression. HC et al. 49 found that miR-206 activates the MEK/ERK and JNK pathways by regulating IRAK1 to promote LPS-induced inflammatory damage. In addition, previous studies have reported that MER/ERK pathways could regulate ETS1 through its phosphorylation at threonine 38 50, 51. Therefore, the effect of KRT80 on the MEK/ERK pathway that we detected may not be independent, but may be a superimposed effect. Meanwhile, we found that miR-206 has no direct effect on KRT80. Therefore, we speculate that miR-206/ETS1 regulates KRT80 to mediate the progression of ovarian cancer through the MEK/ERK pathway.

## Conclusion

In summary, we determined that KRT80 is significantly overexpressed in ovarian cancer and is associated with a poor prognosis. KRT80 overexpression stimulates the MEK/ERK signaling pathway and promotes the proliferation, G0/G1 phase to S phase transition, invasion, and migration of ovarian cancer cells. Mir-206 targets ETS1 to regulate KRT80 expression. We revealed a new cancer-promoting mechanism associated with KRT80. KRT80 is expected to play an important role in the early diagnosis, prognosis evaluation, and clinical treatment of ovarian cancer in the future, and is a promising regulatory gene for ovarian cancer.

## Supplementary Material

Supplementary figure S1.

## Figures and Tables

**Table 1:** Expression of KRT80 in different types of ovarian tissue

Group	Cases	Low expression		High expression		Positive rate (%)	High expression rate (%)
		(-)	(+)	(++)	(+++)		
Malignant	102	21	23	35	23	79.41^a,b^	56.86^c,d^
Borderline	16	4	7	4	1	75.00^e,f^	31.25^g,h^
Benign	14	10	3	1	0	28.57	7.14
Normal	15	11	4	0	0	26.67	0

Note: a Malignant vs. benign (***, *P* < 0.001); b Malignant vs. normal (***, *P* < 0.001); c Malignant vs. benign (***, *P* < 0.001); d Malignant vs. normal (***, *P* < 0.001); e Borderline vs. benign (*, *P* = 0.026); f Borderline vs. normal (*, *P* = 0.012); g Borderline vs. benign (*P* = 0.175); h Borderline vs. normal (*, *P* = 0.043).

**Table 2:** Relationship between KRT80 expression and clinicopathological parameters of ovarian epithelial malignant tumors

Groups	Cases	Low expression		High expression		Positive rate (%)	*P*	High expression rate (%)	*P*
		(-)	(+)	(++)	(+++)				
**Age at diagnosis (years)**									
<53	59	15	13	22	9	74.58	0.157	52.54	0.302
≥53	43	6	10	13	14	86.05		62.79	
**FIGO stage**									
I-II	43	11	15	8	9	74.42	0.287	39.53	0.003**
III-IV	59	10	8	27	14	83.05		69.49	
**Differentiation**									
Well-moderate	52	12	15	17	8	76.92	0.526	48.08	0.068
Poor	50	9	8	18	15	82.00		66.00	
**Lymphatic metastasis**									
No	65	18	18	16	13	72.31	0.049*	44.62	0.002**
Yes	22	1	3	13	5	95.45		81.82	
Unknown^a^	15	2	2	6	5	86.66		73.33	
**Pathological type**									
Serous	67	15	11	26	15	77.61	0.406	61.19	0.184
Mucinous	8	1	3	2	2	87.50		50.00	
Endometrioid	19	5	7	4	3	73.68		36.84	
Clear cell carcinoma	8	0	2	3	3	100.00		75.00	

Note: a 15 patients without lymphadenectomy.

**Table 3:** Univariate and Multivariate Cox Analysis of Different Clinicopathological Parameters with Ovarian Cancer

Variable	Univariate analysis			Multivariate analysis		
	HR	95% CI of HR	*P*	HR	95% CI of HR	*P*
**Age at diagnosis**						
<53	2.047	0.957-4.382	0.065			
≥53						
**FIGO stage**						
I-II	5.904	2.237-15.578	<0.001***	4.183	1.304-13.417	0.016*
III-IV						
**Differentiation**						
Well-moderate	1.126	0.549-2.308	0.746			
Poor						
**Lymphnode metastasis**						
No	3.138	1.381-7.133	0.006**	1.162	0.474-2.853	0.743
Yes						
**KRT80**						
Low	5.627	2.145-14.763	<0.001***	4.198	1.372-12.849	0.012*
High						
