# Supplementary material for: Keratin 80 regulated by miR-206/ETS1 promotes tumor progression via the MEK/ERK pathway in ovarian cancer
Source: J Cancer. 2021 Sep 24;12(22):6835–50. doi: 10.7150/jca.64031 (PMC8517993; doi:10.7150/jca.64031)
Supplement: Supplementary file 1 — Supplementary figure S1. [file jcav12p6835s1.pdf]

Figure S1. Representative images and quantitation of the western blotting showed that the protein expression of KRT80 in the miR-206 mimics/inhibitor groups ($n = 3$). GAPDH was used as an internal control. Data are presented as mean ± SD.

Figure S1

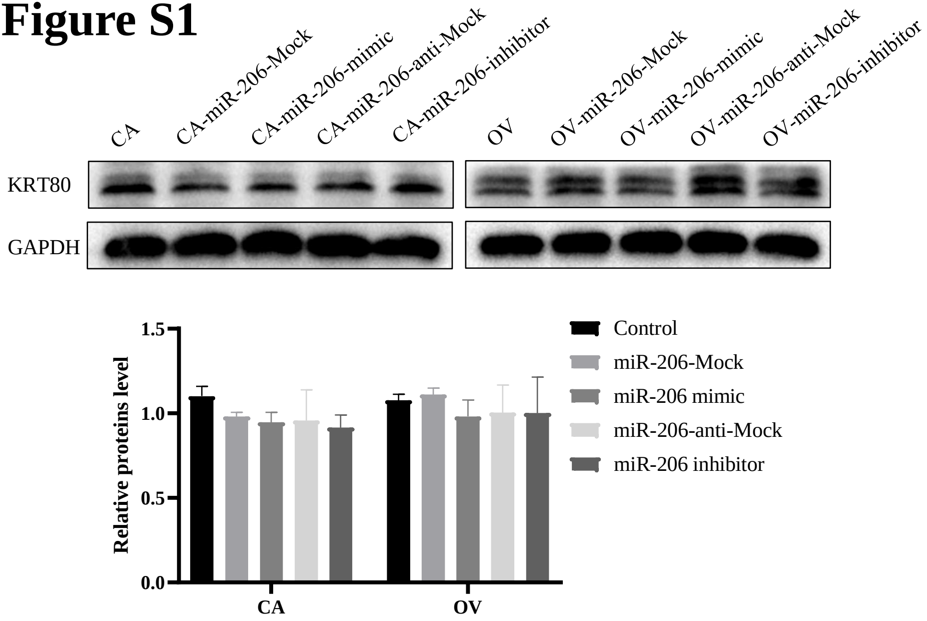
CA
CA-miR-206-Mock
CA-miR-206-mimic
CA-miR-206-anti-Mock
CA-miR-206-inhibitor
OV
OV-miR-206-Mock
OV-miR-206-mimic
OV-miR-206-anti-Mock
OV-miR-206-inhibitor
KRT80
GAPDH
Relative proteins level
1.5
1.0
0.5
0.0
CA
OV
Control
miR-206-Mock
miR-206 mimic
miR-206-anti-Mock
miR-206 inhibitor
